# Comparative Efficacy of Novel Biomimetic Remineralising Technologies

**DOI:** 10.3390/biomimetics8010017

**Published:** 2023-01-02

**Authors:** Peiyan Shen, James R. Fernando, Yi Yuan, Coralie Reynolds, Eric C. Reynolds

**Affiliations:** Centre for Oral Health Research, Melbourne Dental School, Bio21 Institute, The University of Melbourne, Melbourne, VIC 3010, Australia

**Keywords:** CPP–ACP, fluoride, fluoro calcium phosphosilicate, self-assembling P11-4 peptides, enamel subsurface lesions, remineralisation

## Abstract

Biomimetic technologies for the remineralisation of enamel subsurface lesions (ESLs) have been developed and include: fluorocalcium phosphosilicate bioglass (BG/F); casein phosphopeptide-amorphous calcium phosphate (CPP–ACP) and with fluoride (CPP–ACFP); and self-assembling oligopeptide P11-4 (SAP). The aim of this study was to compare the remineralisation of ESLs in vitro using these technologies. Human enamel slabs with ESLs were cut into two half-slabs; one half-slab was untreated (control), and the other half was treated by exposure to one of the four technologies with artificial saliva (AS) or AS alone for 14 days at 37 °C. The technologies were applied to the ESL surface according to the manufacturer’s instructions. At the completion of each treatment, the treated half-slabs and their paired control half-slabs were embedded, sectioned and the mineral content was determined using transverse microradiography. The change in mineral content (remineralisation) between treatments was statistically analysed using one-way ANOVA. The order from highest to lowest remineralisation was CPP–ACFP (52.6 ± 2.6%) > CPP–ACP (43.0 ± 4.9%) > BG/F (13.2 ± 2.5%) > SAP (5.8 ± 1.6%) > AS (2.1 ± 0.5%). Only CPP–ACFP and CPP–ACP produced remineralisation throughout the body of the lesions. All four biomimetic technologies had some effect on the remineralisation of ESLs; however, CPP–ACFP with calcium, phosphate and fluoride ions stabilised by CPP was superior in the level and pattern of remineralisation obtained.

## 1. Introduction

Dental caries is one of the most prevalent chronic diseases and affects individuals of all ages [1]. It is initiated by the demineralisation of tooth mineral by organic acids produced by fermentation of dietary sugars by dental plaque bacteria [2]. It is still a major public health problem worldwide despite a decrease in its prevalence in most developed countries through the use of fluoride-containing oral care products. Fluoride is regarded as an important strategy in managing caries and erosion [3,4,5]. Although fluoride-mediated remineralisation is the foundation of current approaches to caries management, remineralisation of enamel subsurface lesions (ESLs) by fluoride, to form fluorhydroxyapatite [Ca_10_(PO_4_)_6_(OH)_(2-2x)_F_2x_, where x < 1.0], is limited by the bioavailability of calcium ions [6,7,8]. Consequently, the development of new remineralisation therapies providing bioavailable calcium ions has been a priority in the last two decades. The principles of minimally invasive dentistry dictate the need for clinically effective measures to remineralise ESLs, both for structural and aesthetic reasons. ESLs, also known as white spot lesions, on labial surfaces of anterior teeth post-orthodontic therapy, can be an aesthetic issue resulting in patient dissatisfaction, as well as an ongoing concern for progression to cavitation and the need for restoration [9,10]. The prevalence of ESLs among orthodontic patients can be as high as 96% [11,12,13].

The latest advances in remineralisation technologies involve the biomimetics of various elements of the natural mineralisation systems in saliva and enamel formation. These elements are the calcium-stabilising salivary protein-like ion delivery vehicles, and the hydroxyapatite, hydroxyapatite-like or enamel protein-like nucleation templates [14,15]. Examples of the biomimetic technologies related to these elements are casein phosphopeptide-stabilised amorphous calcium phosphate nanocomplexes (CPP–ACP) based on the salivary protein Statherin [16]; bioactive glass BioMin^TM^ F as a hydroxyapatite-like nucleation site and ion-releasing template [17,18,19] and the self-assembling template peptide P11-4 as a biomimetic of amelogenin [20,21]. 

For the CPP–ACP biomimetic technology, CPP contains the sequence -Ser(P)-Ile/Leu-Ser(P)-Ser(P)-Ser(P)-Glu-Glu- which stabilises supersaturated solutions of calcium, phosphate and fluoride ions to produce electroneutral casein phosphopeptide-amorphous calcium fluoride phosphate nanocomplexes (CPP–ACFP) [22,23]. The CPP are biomimetics of the salivary protein Statherin which contains the sequence Asp-Ser(P)-Ser(P)-Glu-Glu-; however, due to the greater content of Ser(P), CPP is claimed to be superior to Statherin in is ability to stabilise and deliver calcium, phosphate and fluoride ions to remineralise ESLs [8]. 

The BioMin^TM^ bioglass biomimetic mineralisation template technology is based on fluoro calcium phosphosilicate which is claimed to diffuse into the ESLs, to release ions and provide a hydroxyapatite-like nucleation site for the formation of fluorhydroxyapatite [17,18,19,24]. Finally, the amelogenin-biomimetic technology P11-4 peptide (Curodont^TM^ Repair) has also been claimed to diffuse into ESLs and act as a self-assembling template for remineralisation [25]. The self-assembling peptide P11-4 has the amino acid sequence: Ace-Gln-Gln-Arg-Phe-Glu-Trp-Glu-Phe-Glu-Gln-Gln-NH_2_ which exhibits similar physicochemical properties to amelogenin-derived peptides that act as templates for the mineralisation of enamel [26,27]. It is reported that the P11-4 fibrillar matrix has a high affinity for Ca^2+^ ions and acts as a nucleator for de novo hydroxyapatite (HA) formation resulting in remineralisation of the lesion body [27,28].

While the development of novel biomimetic remineralisation systems has progressed significantly in recent years, BioMin^TM^ F and Curodont^TM^ Repair are new oral care products and there is insufficient independent evidence to assess their true clinical potential. Hence, the aim of this project was to compare the in vitro efficacy of (i) Tooth Mousse (TM) containing CPP–ACP, (ii) Tooth Mousse Plus (TMP) containing CPP–ACFP with 900 ppm F, (iii) BioMin F containing fluoro calcium phosphosilicate with 590 ppm F, and (iv) Curodont Repair containing self-assembling peptide P11-4, to remineralise enamel subsurface lesions. The null hypothesis for the study was that no significant difference in lesion mineral content or lesion depth would be detected after treatment with each of these technologies.

## 2. Materials and Methods

### 2.1. Biomimetic Remineralisation Products

Four commercially available oral care products containing the biomimetic remineralisation technologies were tested (Table 1). The four products were: (1) TM (GC, Japan) containing CPP–ACP; (2) TMP (GC, Tokyo, Japan) containing CPP–ACFP with 900 ppm F; (3) BioMin F (BioMin Technologies Ltd., London, UK) containing fluoro calcium phosphor silicate with 590 ppm F (BG/F); (4) Curodont^TM^ Repair (Credentis, Windisch, Switzerland) containing self-assembling P11-4 peptides (SAP).

### 2.2. Enamel Subsurface Lesion Preparation 

**Figure 1:**
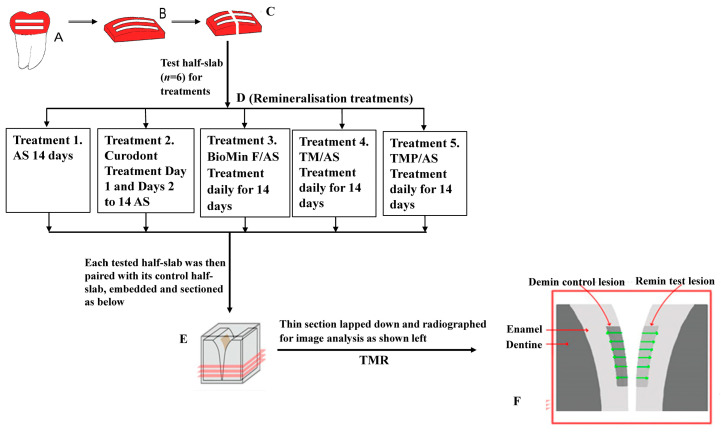
Schematic showing enamel subsurface lesion (ESL) preparation, sectioning of control and treatment half-slabs, remineralisation treatments, pairing of treatment and control half-slabs for sectioning and TMR analysis. A, B and C (Lesion preparation): Extracted human third molars were polished and painted with nail varnish to form 2 mesiodistal windows (A). Enamel slabs (B) were sawn from the painted aspect of each tooth. After demineralisation, slabs were cut perpendicular to the windows into test half-slabs (left) and control half-slabs (right) (C). D, E and F: Test half-slabs (*n* = 6) were tested with the five different treatments as indicated (D). Tested half-slabs were then paired with their control half-slabs, embedded, sectioned (E) and radiographed for image analysis (F).

Extracted intact non-carious human third molars were obtained from private dental practices in Melbourne, Australia, after obtaining informed patient consent. The study was approved by the University of Melbourne’s Human Research Ethics Committee (Approval no. 12666). The teeth were sterilised by storage for at least 14 days in 10% (*v*/*v*) neutral buffered formalin solution at room temperature. Enamel slabs were cut from the sterilised teeth and subsurface lesions were created as previously described [29]. This involved painting the enamel slabs with acid-resistant nail varnish (Red 745, Revlon, Oxford, NC, USA) to expose two windows (1 × 7 mm) of enamel on each slab that were demineralised for four days at 37 °C using a demineralisation buffer to produce subsurface lesions of approximately 100 μm depth [29]. The composition of the demineralisation buffer was 80 mL/L Goodrite K-702 polyacrylate (Lubrizol Advanced Materials Inc., Cleveland OH, USA), 500 mg/L hydroxyapatite (Bio-Gel HTP, Bio-Rad Laboratories, Hercules, CA, USA), and 0.1 M lactic acid (Ajax Chemical, Mt Pritchard, NSW, Australia), adjusted to pH 4.8. The enamel slabs containing subsurface lesions were cut into two half-slabs. One of the half-slabs was retained as the demineralisation control and the other half-slab (test half-slab) was exposed to one of the four products in artificial saliva (AS) or to AS alone and subsequent remineralisation was measured (see below and Figure 1).

### 2.3. Remineralisation Protocol

The remineralisation model used was an in vitro simulation of the remineralisation of existing ESLs after post-orthodontic therapy where the plaque-retaining bracket has been removed and the porous white spot lesions are able to be remineralised by application of a remineralisation technology. Thirty enamel test half-slabs were randomly allocated to one of the five experimental groups. Six half-slabs each containing two demineralised subsurface lesions were used for each of the five treatments: (1) Artificial saliva (AS) alone; (2) Curodont (SAP) + AS; (3) BioMin (BG/ F) + AS; (4) TM + AS; (5) TMP + AS. 

Curodont (SAP) was directly applied to the lesion surfaces on the enamel test half-slab (one application per test half-slab) and allowed to diffuse into the lesion for 5 min (to closely follow the manufacturers’ recommendations) before being suspended in 4 mL AS. For TM, TM Plus and BioMin groups, enamel half-slabs were each suspended in 1 g of the test product dissolved in 4 mL AS once per day, as per the manufacturer’s instructions. For the negative control group enamel half-slabs were suspended in 4 mL of AS. AS consisted of 50 mM NaCl, 0.5 mM CaCl_2_ and 0.5 mM Na_2_HPO_4_/NaH_2_PO_4_ pH 7.0. Each half-slab was coded and when not being treated were suspended unagitated in AS at 37 °C. The treatment period was for 14 days. The solutions were replaced with freshly prepared solutions each day during the 14-day period. At the end of the 14-day period, each test half-slab was removed, rinsed with ethanol to remove the nail varnish and washed thoroughly in distilled deionised water (DDW), blotted dry, then paired with its respective control (i.e., demineralised half-slab) for sectioning.

### 2.4. Sectioning and Transverse Microradiography

Each test half-slab paired with its corresponding control half-slab, coded (blinded) was embedded, sectioned, and analysed by transverse microradiography (TMR) to determine mineral content as described previously by Shen et al. [29]. Briefly, each section, which contained the remineralised half-lesion and the paired demineralised control half-lesion from the same enamel slab, was radiographed along with an aluminium step wedge of 7 × 37.5 µm thick increments using Microchrome High-Resolution glass plates (HTA Enterprises, Microchrome Technology Products, San Jose, CA, USA) and nickel-filtered Cu Kα radiation at 35 kV, 20 mA for six minutes using a custom-filtered TMR system (Diffraction Technologies, Mt Eliza, VIC, Australia). The X-ray source was a glass 1200W PANalytical fine-focus tube with a Cu target (PANalytical, Condell Park, NSW, Australia). The X-ray tube was powered by a Spellman XLF −60 N 1200 generator (Spellman HV, Hauppauge, NY, USA) cooled with recirculated and refrigerated water using a Polyscience water chiller model 6706P (Polyscience, Niles, IL, USA). The Cu Kβ radiation was attenuated using a 15 μm Ni filter. 

The depth of the control demineralised lesion was represented as LDd, and the depth of the treated lesion was represented as LDr. The vol% mineral profile of each enamel slab’s demineralised control and treated lesion was compared with the sound enamel % mineral profile of the same section. The difference between the area under the densitometric profile of the control lesion and the sound enamel, calculated by trapezoidal integration, was represented by ΔZd. The difference between the area under the densitometric profile of the treated lesionand the sound enamel, calculated by trapezoidal integration, was represented by ΔZr. These parameters were then used to calculate % Remineralisation (%R) using the formula:


$$\%R\;=\;\frac{\Delta \mathrm{Zd}\;-\;\Delta \mathrm{Zr}}{\Delta \mathrm{Zd}}\;\times \;100$$

### 2.5. Statistical Analysis

Means and standard deviations for lesion parameters associated with each of the five groups using the slab as the experimental unit were calculated. Differences in both LDd and ΔZd across the five groups were measured using one-way analysis of variance. Differences in the change in mineral content of lesions (ΔZd − ΔZr) across the five groups were measured using univariate analysis of covariance with ΔZd as the covariate on log transformed ΔZd − ΔZr values. Differences between mean ΔZd − ΔZr values for each treatment were measured using pairwise comparisons with a Šidák adjustment for multiple comparisons. Differences in %R of lesions for each treatment were measured using univariate analysis of variance on square-root-transformed values. ΔZd was not included as a covariate as its effect was not significant (*p* > 0.05). Differences between mean %R values for each treatment were measured using pairwise comparisons with a Šidák adjustment for multiple comparisons. Normality of residuals was tested with the Shapiro–Wilk test and normal Q–Q plots. Homogeneity of variance of the residuals was confirmed using Levene’s test. Statistical analyses were performed using IBM SPSS Statistics for Windows, version 26 (IBM Corp., Armonk, NY, USA) using a significance level of α = 0.05. Box–Cox transformations were performed using Minitab 19 Statistical Software (Minitab, Inc., State College, PA, USA).

## 3. Results

### 3.1. Mineral Content Change after Treatment

**Figure 2:**
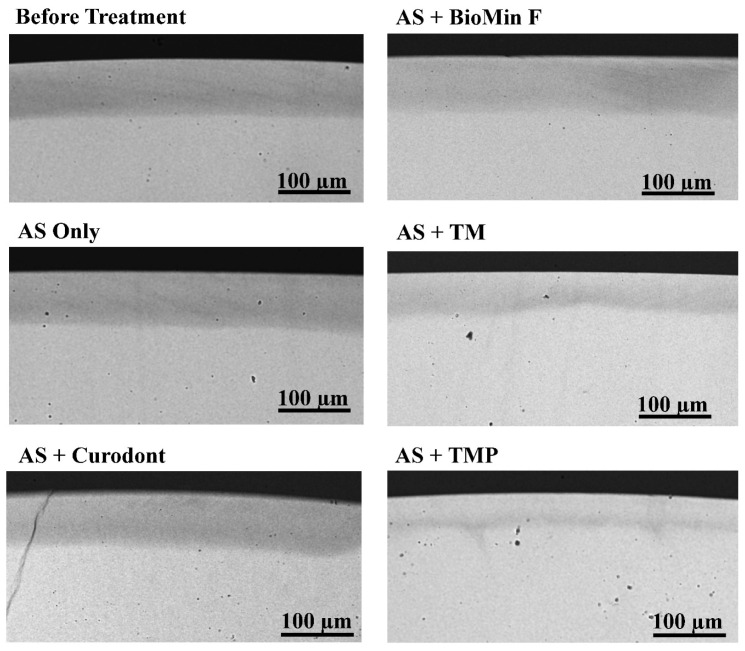
Representative microradiographic images. Representative images of transverse microradiographs of a demineralised lesion before treatment and five remineralised lesions after treatment with AS Only, AS + Curodont, AS + BioMin, AS + TM and AS + TMP. The scale bar on each image represents 100 µm.

The effect of the four different products (BioMin, Curodont, TM and TMP) in AS on the remineralisation of ESLs compared with AS alone is shown in Table 2. Representative images of transverse microradiographs before and after treatment with AS Only, AS + Curodont, AS + BioMin, AS + TM and AS + TMP, are presented in Figure 2. The ESLs prepared for the remineralisation study were uniform and there were no significant differences in either mineral content (ΔZd) or lesion depth (LDd) of the initial demineralised lesions across each of the five treatments before remineralisation treatment commenced (Table 2). 

However, significant differences were observed in mineral content of the lesions after the different remineralisation treatments. As shown in Table 2, there was a significant overall difference in the mean mineral content change (ΔZd − ΔZr) following treatment with the AS only and the four products (*p* < 0.0001), with mean ΔZd − ΔZr values ranging from 44.6 ± 12.2 vol%min.µm (AS only) to 1168.6 ± 112.7 vol%min.µm (TMP). The mean ΔZd − ΔZr values for TM and TMP were not significantly different (*p* > 0.05), but both were significantly greater than the mean ΔZd – ΔZr values following treatment with AS alone, Curodont and BioMin (*p* < 0.05). 

TMP produced the highest mean percent remineralisation (%R) of the ESLs (Table 2); this was significantly higher than the mean %R values for all the other treatments (*p* < 0.05). The mean %R values for the five groups ranged from 2.11 ± 0.47% (AS only) to 52.64 ± 2.56% (for TMP containing CPP–ACFP). The mean %R value for TMP containing CPP–ACFP was significantly higher than for TM containing CPP–ACP (*p* < 0.05). Mean %R values for both TMP and TM were significantly greater than for BioMin and Curodont (*p* < 0.001). The order of the level of remineralisation following the five treatments from lowest to highest was: AS Alone < Curodont < BioMin < TM < TMP.

On a proportional basis, TMP produced 22.4% greater remineralisation than TM, 297.9% greater remineralisation than BioMin F, and 815.5% greater remineralisation than Curodont (Table 2).

### 3.2. Enamel Subsurface Lesions Depths

There were no significant differences in changes in mean lesion depths (LDd−LDr) following treatment with TM (22.09 ± 6.46 µm) and TMP (33.23 ± 10.73 µm) (*p* > 0.05), although there was a clear trend of a greater effect with TMP, as shown in Figure 2. However, these values were both significantly greater than the mean LDd−LDr values for BioMin, Curodont, and AS only (*p* < 0.05). There were no significant differences between mean LDd−LDr values for BioMin, Curodont, and AS alone (*p* > 0.05) (Table 2).

## 4. Discussion

This study demonstrated that all the biomimetic remineralisation technologies had some effect on returning lost mineral to the ESLs such that the null hypothesis was rejected. However, it was clear that the saliva biomimetic technology based on CPP–ACP and CPP–ACFP in TM and TMP, respectively was superior to the hydroxyapatite-like BioMin technology and the amelogenin-like self-assembling peptide template technology Curodont, in their abilities to remineralise ESLs. The superior remineralisation by TMP is attributed to the presence of the CPP–ACP and fluoride. CPP–ACP, in the presence of sodium fluoride, has been shown to form CPP–ACFP nanocomplexes [22,30,31]. These nanocomplexes have a hydrodynamic radius of 2.12 ± 0.26 nm [22] and are electroneutral ion clusters allowing rapid diffusion deep into the ESLs through the nanoporosities of the surface layer [16]. The combination of CPP–ACP and fluoride to form CPP–ACFP nanocomplexes has been shown to be superior to fluoride alone in inhibiting enamel demineralisation and promoting remineralisation of ESLs in a number of randomised, double-blind clinical trials [32,33,34,35,36]. 

CPP is a saliva biomimetic but with a significantly greater calcium-stabilising capacity than salivary proteins due to the higher content of its phosphoseryl residues [8]. CPP-amorphous calcium phosphate (ACP) nanocomplexes are readily soluble in saliva and exhibit a high affinity for the tooth surface where they can maintain an ion diffusion gradient into ESLs to promote remineralisation deep in the lesion. The subsurface remineralisation pattern produced by CPP–ACP has been shown to significantly improve the aesthetics, strength, and acid resistance of the remineralised lesion [16,37]. CPP–ACP is probably the most widely studied non-fluoride remineralising agent; many randomised clinical trials have demonstrated that CPP–ACP products have significantly greater remineralising and anticariogenic properties than placebos or products based on fluoride alone [8,32,33,38,39,40,41,42,43,44].

BioMin was introduced on the market quite recently but is similar to other bioglass products. It has been claimed that bioglass is a mimetic of hydroxyapatite, thus catalysing the formation of fluorapatite in ESLs, thereby remineralising lost tooth structure. BioMin, similarly to the other bioglasses, contains fluoro calcium phosphosilicate with an available fluoride content of 590 ppm. The level of ESL remineralisation following BioMin treatment in this study was very modest at 13.2 ± 2.5 %. This observed remineralisation is likely to result from the low level of soluble fluoride ion release from the fluoro calcium phosphosilicate when added to the AS [21].

Recently, the self-assembling peptide P11-4 technology was evaluated for its ability to remineralise and, as a consequence, mask artificially created ESLs [45]. The authors concluded that the self-assembling peptides could neither remineralise nor mask the lesions [45]. The results of the present study are consistent with this previous finding; however, the greater sensitivity of the methodology used in the current investigation did show a very low, but significant, increase in remineralisation of ESLs by the Curodont product containing the self-assembling peptide technology. This very low increase in remineralisation may be partly attributed to the high pH and buffering capacity of Curodont driving remineralisation as pH modulation has recently been shown to help accelerate ESL remineralisation [46].

Both biomimetic technologies based on providing nucleation templates inside the ESL, BioMin bioglass providing a hydroxyapatite-like nucleation site and Curodont self-assembling amelogenin-like peptide, failed to produce high levels of ESL remineralisation. It is not too surprising that ESLs do not need to be provided with intra-lesion nucleation sites to effect remineralisation as they consist of partially demineralised apatite crystals which act as the mineralisation template. ESLs have only normally lost 30–40% of their mineral content with a relatively intact surface layer. The demineralised crystals are characterised by a loss of ions usually starting at the carbonated crystal centres which can be remineralised if supplied with an excess of bioavailable calcium, phosphate, and fluoride ions [16]. Saliva has the ability to remineralise these early lesions due to the level of bioavailable calcium phosphate and fluoride ions stabilised by salivary phosphopeptides. The salivary biomimetic CPP–ACFP rapidly dissolves to substantially enhance saliva’s ability to remineralise ESLs as demonstrated in this in vitro study.

The results of this in vitro study on the remineralisation efficacy of the other biomimetic technologies needs to be assessed in clinical trials as has been conducted for the CPP–ACP technology. [32,33,47,48].

## 5. Conclusions

In conclusion, fluoride-mediated remineralisation is the foundation of management of ESLs, but this is limited by the bioavailability of calcium. Biomimetic technologies have been developed which help promote fluoride remineralisation but the phosphopeptide-stabilised calcium phosphate technologies, which are a biomimetic of saliva’s remineralisation system, appear to have the most promise. This in vitro study showed that calcium, phosphate, and fluoride ions stabilised in CPP–ACFP nanocomplexes were superior in promoting remineralisation of ESLs, with CPP facilitating the uptake of bioavailable calcium, phosphate, and fluoride ions deep into the lesions.

## Figures and Tables

**Table 1:** List of products tested.

Product Name	Biomimetic Technology
Tooth Mousse^®^ (GC, Tokyo, Japan)	10% (*w*/*w* CPP–ACP
Tooth Mousse Plus^®^ (GC, Tokyo, Japan)	10% (*w*/*w*) CPP–ACFP with 900 ppm F
BioMin^TM^ F (BioMin Technologies Ltd., London, UK)	Fluoro Calcium Phosphosilicate with 590 ppm F
Curodont^TM^ Repair (Credentis, Windisch, Switzerland)	Self-assembling P11-4 peptides

**Table 2:** Remineralisation of enamel subsurface lesions by different biomimetic technologies in artificial saliva (AS).

Treatment	LDd (µm)	LDd−LDr (µm)	∆Zd (vol%min.µm)	∆Zd − ∆Zr (vol%min.µm)	%R
AS only	94.33 ± 5.66 *	−3.01 ± 5.44 ^ab^	2059.11 ± 92.06	44.61 ± 12.20 ^abcd^	2.11 ± 0.47 ^abcd^
AS + Curodont	84.54 ± 6.63	−0.45 ± 7.73 ^cd^	2171.12 ± 301.86	126.20 ± 46.14 ^aefg^	5.75 ± 1.63 ^aefg^
AS + BioMin F	87.26 ± 7.49	4.30 ± 5.29 ^ef^	2149.35 ± 262.14	286.53 ± 78.08 ^behi^	13.23 ± 2.45 ^behi^
AS + TM	92.97 ± 5.65	22.09 ± 6.46 ^ace^	2577.04 ± 246.75	1113.83 ± 215.53 ^cfh^	43.00 ± 4.94 ^cfhj^
AS + TMP	93.62 ± 7.30	33.23 ± 10.73 ^bdf^	2228.64 ± 267.49	1168.58 ± 112.70 ^dgi^	52.64 ± 2.56 ^dgij^

* Mean ± Standard Deviation. LDd: *p* > 0.05. ∆Zd: *p* > 0.05. LDd-LDr: ^e^ *p* < 0.05, ^c^ *p* < 0.01, ^a^ *p* < 0.001, ^bdf^ *p* < 0.0001. ∆Zd − ∆Zr: All treatments were significantly different (*p* < 0.0001) except AS + TM and AS + TMP (*p* > 0.05). ^abcdefghi^ *p* < 0.0001. %R: ^j^ *p* < 0.05, ^a^ *p* < 0.001, ^bcdefghi^ *p* < 0.0001.

## Data Availability

The authors confirm that the data supporting the findings of this study are available within the article.
